# Human Development and Pastoral Care in a Postmodern Age: Donald Capps, Erik H. Erikson, and Beyond

**DOI:** 10.1007/s10943-017-0483-0

**Published:** 2017-09-13

**Authors:** Hetty Zock

**Affiliations:** Faculty of Theology and Religious Studies, University of Groningen, Oude Boteringestraat 38, 9712 GK Groningen, The Netherlands

**Keywords:** Donald Capps, Erik H. Erikson, Human development, Postmodernity, Pastoral care, Hermeneutical psychology

## Abstract

This article discusses Donald Capps’s use of Erik H. Erikson’s life-cycle theory as the basic psychological framework for his theory of pastoral care. Capps was attracted to Erikson’s existential-psychological model, his hermeneutic approach, and his religious sensitivity. Capps’s thought develops from first exploring biblical foundations for using Eriksonian theory for pastoral care to gradually embracing certain postmodern features. The article concludes with reflections on the usefulness of Erikson’s life-cycle theory and Capps’s work for contemporary pastoral care.

A text becomes a classic because readers find it personally meaningful to them and … it remains a classic because readers discover over the course of their lives new ways in which it is meaningful to them.

—Capps (2015a, p. 328) on *Young Man Luther*

## Meeting Donald Capps

I came across the name of Donald Capps for the first time while doing my doctoral research on Erik H. Erikson. Richard Hutch, an Australian colleague in psychology of religion, suggested that I read Capps’s article “Erikson’s Life-Cycle Theory: Religious Dimensions” (Capps 1984). I was excited to read it; this was precisely the angle for which I had been looking. Capps touched upon the very issue that fascinated me but was struggling to articulate, namely the elusive but fundamental role of religion in Erikson’s psychology.

Like Capps, I had been captured by Erikson’s *Young Man Luther*. It was the first book I read during my studies in psychology of religion in which religion was not reduced to bodily, psychic, social, or cultural factors but instead analyzed as a distinct yet intricately related dimension of human development. In his psychohistorical studies of *homines religiosi*—great religious figures such as Luther, Gandhi, and Jesus—Erikson elaborately shows how the existential-religious, the psychosocial, and the cultural-historical dimensions of reality interconnect in human development. For this reason, I chose to focus in my doctoral research on Erikson’s life-cycle theory as an analytic hermeneutical tool in psychology of religion. How, I wondered, do existential-religious and psychosocial development interact in shaping in human lives and societies? In his rich 1984 article, Capps showed that Erikson’s life-cycle theory is a religious construct in the sense that Erikson increasingly incorporated existential and religious language in his life-cycle theory. I elaborated on this basic idea in my book on Erikson (Zock 1990/2004), in which I argue that the often overlooked existential-religious dimension of his work permeates his developmental theory.

It therefore is a great pleasure and honor to contribute to this special issue, by reflecting on four of Capps’s nearly countless works in which Erikson’s life-cycle theory plays a central role: *Life Cycle Theory and Pastoral Care* (1983), *Deadly Sins and Saving Virtues* (1987), *The Decades of Life* (2008), and *Still Growing* (2015b). Erikson’s work, and in particular his life-cycle theory, has been a theoretical and inspirational foundation for Capps as a pastoral theologian from his earliest to his very last publications.

Capps and I shared a love for Erikson’s work: for its positive and dynamic view on human growth; its sensitivity to the spiritual and religious aspects of human life in ever changing forms and contexts; its artistic, sensitive, and sensible analyses of human phenomena; and its deeply personal, contextual, and psychohistorical approach. Starting from a Freudian ego psychological perspective, Erikson developed a theory of personality development that is intrinsically psychosocial. To understand human phenomena, according to Erikson, one must always take three interdependent processes into account: *soma*—i.e., bodily, organismic aspects; *psyche*—the organizing of individual experience by the ego; and *ethos*—the relational, communal, and cultural processes (Erikson 1982, pp. 25ff.). This contextual and cultural approach makes Erikson’s psychology applicable *par excellence* to pastoral care, which takes place in the midst of everyday human life.

As an Eriksonian scholar myself discussing the meaning and value of Capps’s use of Erikson’s life-cycle theory, I hold that our own personal contexts as scholars matter. Erikson argues that theories and research become meaningful only in specific contexts for specific researchers who find the research meaningful for their own lives. So, it is important to reflect both on Capps’s creative, manifold, and continually evolving uses of the life-cycle theory in his pastoral theological writings and on my own uses of it here. This is not a narcissistic endeavor (though Capps would be attentive to narcissistic motives) but an appropriate way of working “along Eriksonian lines.”

I met Donald Capps in person only a few times on visits to Princeton Theological Seminary. A vivid image of him emerges: as a tall, lean figure wandering the grounds of Princeton Theological Seminary—not taking the paved paths but walking right across the lawns—and as a gracious and modest host and moderator. But I have the feeling I know him quite well, for his work has such a personal touch. His writing drew on case studies and his own experiences and reflections. Capps was a leading figure in American Protestant pastoral theology, rooted in the Evangelical Lutheran denomination, in the last two decades of the twentieth and the first decade of the twenty-first centuries. He was very much aware of the changes in American society and church life and used Erikson’s life-cycle theory to develop a dynamic theory of pastoral care and counseling.

My context is a different one. I am a liberal female Protestant theologian and psychologist of religion, a generation—twenty-some years—younger than Capps. I have worked as a pastor in the liberal wing of the Dutch Reformed Church for 14 years and have taught psychology of religion and spiritual care for 25 years. I am living and working in an utterly secularized country, teaching “non-denominational spiritual care” (Zock 2008, 2010). Only one-third of the Dutch population has an official religious affiliation. Religion is to a high degree deinstitutionalized, and young people are no longer familiar with the Christian tradition. For instance, the majority could not tell the precise meaning of Easter as a Christian feast. But the interest in “spirituality” and the search for meaning nonetheless remains huge. The churches and chaplains in public institutions embrace the task of developing a new language that is not explicitly religious for counseling, supporting, and orienting persons in life, illness, and death. I was drawn to Erikson’s work because I recognized in it a sensitivity to existential issues underlying manifold and diverse religious expressions and traditions. It offers a model of existential development that could be helpful for pastoral care in my secular and pluralistic context, a psychology of ultimate concern captured in an existential language that might transcend—and thus hold together—traditional religious languages and free-floating spirituality.

So, my cards are on the table. In the following discussion of Capps’s use of Erikson’s life-cycle theory, I will focus on its meaning for pastoral care in a changing, pluralistic, secular context. I will proceed as follows. First, I will consider Erikson’s model of existential development, as this is the basic psychological framework for Capps’s theory of pastoral care. Then, I will sketch why Capps may have been attracted to Erikson’s work, by illustrating some characteristics of both Erikson’s and Capps’s approaches. Next, I will discuss how Capps creatively uses and adapts Erikson’s life-cycle theory in Capps’s four works on pastoral care mentioned above. I will conclude with some reflections on the usefulness of Erikson’s life-cycle theory for contemporary pastoral care.

## The Existential Dimension of Human Development

Erikson’s life-cycle theory is undisputedly a “classic” and influential theory in developmental psychology, mentioned in every handbook in the field. Erikson is acknowledged as having originated the first life-span theory of development, extending development into old age. Moreover, his description of the eight stages, each focused on a psychosocial task, is widely cited. His concepts of “basic trust” as the cornerstone of development, and of the “identity crisis”—identity being a central issue in modern and postmodern times—have been especially influential. The existential-religious character of his theory, however, is not generally acknowledged.

In my study on Erikson—inspired, as noted, by Capps’s article on life-cycle theory as a religious construct—I have shown that Erikson’s work may be characterized as a “psychology of ultimate concern.” I argue that Erikson’s view on religion is essential to understanding the very nature of his psychology, which may be characterized as a kind of existential psychology. It started with Erikson’s intuition that there is something “more” in human development than instincts, drives, and psychic mechanisms. We recognize this already in the names of the eight psychosocial tasks, considered on a dynamic spectrum between positive (“syntonic” or integrating) and negative (“dystonic” or disintegrating) poles, described in Erikson’s very first formulation of the life-cycle theory (Erikson 1950/1984): basic trust versus mistrust; autonomy versus shame and doubt; initiative versus guilt; industry versus inferiority; identity versus role confusion; intimacy versus isolation; generativity versus stagnation; and ego integrity versus despair. This terminology has a clear existential ring. All bodily, organismic aspects have “a certain lasting existential value,” as Erikson would phrase it in *The Life Cycle Completed* (Erikson 1982, pp. 40ff.). For instance, the way a baby is fed conveys to the child the values of a family and a specific society: “You can drink when you want, there is plenty of food, life is good,” or “You can only get milk at fixed times, it is bad to be so eager.” In the early 1960s, Erikson elaborates on this “more” by linking “virtues”—basic human strengths—to the eight stages: hope, will, purpose, competence, fidelity, love, care, and wisdom. Each stage, he argues, is “endowed with a total quality which we might term ‘animated’ or ‘spirited’” (Erikson 1964, p. 112; Zock 1990/2004, pp. 68ff.). Concomitant to the virtues of each stage are “ritualizations of everyday life,” of which the great religious rituals are specific expressions. Erikson’s theoretical analysis of the existential dimension of human development culminates in his concept of “I,” described as “the observing center of awareness and volition, which can transcend and must survive the psychosocial identity” (Erikson 1968, p. 135).

Erikson’s concept of *I* is influenced by William James; it concerns the introspective capacity and has a mystical and existential flavor. To speak of *I* is a way to confront one’s own “nothingness”; the search for our origins takes us back before our earliest parental images to “the unborn core of creation, the—as it were, preparental—center where God is pure nothing: *ein lauter Nichts*, in the words of Angelus Silesius” (Erikson 1958, p. 264; Capps 1984, p. 121). It is in this confrontation with one’s own nothingness in which, paradoxically, an existential sense of self—a sense of *I*—arises as a vertical anchoring of horizontal psychosocial development in time and space. Here, a meeting with the “ultimate” takes place—personalized, for Erikson, in the numinous experience of looking another in the eye, as does a motherly caretaker in greeting her baby. This existential meeting with the ultimate, however, does not take place in an isolated domain but is always anchored in, and dependent for its realization on, the psychosocial development in time and space of concrete human lives in specific societal and cultural contexts. “The ultimate resides in the immediate” (Erikson 1975, p. 247).

Erikson developed a new psychological terminology to capture existential dimensions of human development, a language that pairs Freudian ego psychology of bodily impulses with ego synthesis. This leads to a theory highlighting the interdependence of existential and psychosocial aspects of the development of ego and self. In this we find the psychological infrastructure that Erikson employs in his studies of great religious figures.

Capps began studying and applying Erikson’s psychohistorical concepts early in his scholarly career. He concluded that Erikson’s work increasingly demonstrates a religious psychology, its existential dimension taking a specifically religious tone. Capps found Erikson’s life-cycle theory to reflect the religious ideas of the very *homines religiosi* of whom Erikson wrote. For Capps, this made Erikson’s work suitable as a foundation for his pastoral theological models, as I will show below. In my own work, I have continued over the years to look for even broader ways to use Erikson’s ideas to transcend concrete religious traditions and to analyze existential development in a secular and multicultural world.

## Three Reasons Undergirding Capps’s Attraction to Erikson’s Work

### Introspection in the Service of Sanity

Both men endeavored to increase the margin of man’s inner freedom by introspective means applied to the very center of his conflicts; and this to the end of increased individuality, sanity, and service to men (Erikson 1958, p. 252).

This is what Erikson, in his seminal study on the young Luther, notes about Luther and Freud. But it is as telling a characterization of Erikson himself and, I would suggest, of Donald Capps as well. Capps would not have characterized his own life’s work so grandiosely, as a great mission. But, in my view, this purpose gets to the heart of what he tried to establish as a pastoral theologian and what appealed to him in Erikson’s work. An Eriksonian approach leads to taking seriously the vicissitudes of psychic life, including problems and derailments. Pastors, Capps believed, should pay attention to these vicissitudes in order to promote individual and societal wellbeing. Introspection, attentive to psychoanalytic concerns, serves as the royal way.

Capps, like Erikson, was an introspective, somewhat shy, artistically-minded man. He was sensitive to troubled layers in human experience both within himself and others. He did not shy away from bringing into his scholarly work, for instance, unfulfilled longings and disappointments in relation to his mother, who, he discovered later in his life, had wanted him to be a girl. He tells of how this revelation reframed his understanding of his childhood and of how he dealt with and transformed it in his life and work (Capps 2015a).

### A Hermeneutic Approach

I identified with Freud, then, not so much as the former laboratory worker who insisted on a terminology made for the observation of transformable quantities of drive enlivening inner structures, but as the discerner of verbal and visual configurations which revealed what consciousness wanted to enlarge upon, and what it attempted to disguise—and revealed (Erikson 1975, pp. 39ff.).

The hermeneutic character of Erikson’s work also appealed to Capps. Erikson’s approach originated in psychoanalysis and is firmly rooted in the human sciences rather than in the social sciences. He clearly feels more at home with what he calls the “phenomenological, literary” Freud, who is interested in the *meaning* of human phenomena. Erikson’s work therefore belongs, in my view, to the hermeneutical strand of psychology of religion (Zock 1997). It is the meaningful structure of human reality that is the focus of his investigation. Erikson, however, does link biology and hermeneutics; bodily experiences (our bodily hardware, such as epigenetic patterns) do play a role, but these only take shape and meaning in a sociocultural context. This is why we always need interpretative tools (Zock 1997, pp. 131; 140ff.).

Such an approach suited Capps, who majored in English literature and philosophy before concentrating in pastoral theology (Dykstra and Carlin 2016). But it also perfectly suits other theologians and pastors, who are, after all, primarily trained in the humanities. The very task of the pastor involves the reading of “living human documents” (Gerkin 1984), as a still dominant narrative frame in pastoral care puts it.

### Actuality as the Focus of Psychological Analysis

I believe that we can undo this straitjacket [the Cartesian separation between inner and outer worlds] only by separating from our concept of *reality* one of its more obscure implications, namely *actuality*, the world verified in immediate immersion and interaction (Erikson 1964, p. 164).

In his psychological analyses Erikson wanted to grasp not so much factual reality but what he calls “actuality,” i.e., the immediate world of participation as experienced by an active subject who interacts with the environment. His focus was on how individuals and environment affect each other—what those effects are, what they bring about. The criterion for “actuality” is whether or not individual and collective development is furthered—whether “mutual activation” takes place (Zock 1997, p. 143). For instance, in developing a new theology and thus addressing an important question of Renaissance humanity, namely how to become autonomous, Luther confronted at the same time aspects of his own inner turmoil—obstipation, a lack of basic trust, an absent mother and demanding father.

It was this psychosocial principle—the dynamic interplay of soma, psyche, and ethos—that formed the basis of Erikson’s thinking about actualization, but the concept was fully developed only in his later studies on *homines religiosi*. Actualization is in Erikson’s view a religious process. Every *real* actuality, leading to a maximum of actualization and mutual activation, is anchored in an encounter between the *I* and the Ultimate Other (Erikson 1969, pp. 396ff.; Zock 1997, pp. 146ff.). Although actuality is not necessarily achieved in an explicitly religious way, Erikson’s conceptualization of actuality is impacted by the very substances of the religious traditions he studies. We recognize Luther’s deeply personal, existential-mystical faith and Gandhi’s paradoxical nonviolent actions.

Capps notes the importance of actualization and its connection to religion in Erikson’s work (Capps 1996).^1^ He even characterizes Erikson as an “American Actualist,” recognizing in Erikson’s theory of actuality another influential figure, the American poet and philosopher Ralph Waldo Emerson, as someone aligned with Erikson’s depiction of the German Luther and the Hindu Gandhi. Capps sees resemblances between Emersonian themes and the later “religious” Erikson, for whom self-transcendence plays such a great role.

In the following discussion of Capps’s use of life-cycle theory, we will see how these two elements of actuality appear in his theory of pastoral care: in his focus on mutual activation (establishing positive change); and in the religious nature of Erikson’s theory.

## Erikson’s Life-Cycle Theory as the Psychological Frame of Capps’s Pastoral Theology

### Pastoral Care and Life Orientation

In *Life Cycle Theory and Pastoral Care* (1983), which Capps wrote in his early forties, he put forward the outlines of his theory of pastoral care on the basis of Erikson’s life-cycle theory. He would continue to develop and change it, taking into account societal and cultural changes, new academic theories and insights, and his own interests. In his earlier *Pastoral Care: A Thematic Approach* (1979), Capps had already used life-cycle theory to determine the focus of pastoral care—establishing positive change on the personal and the institutional level. But in *Life Cycle Theory and Pastoral Care* he takes a next step in using precisely those elements of Erikson’s theory that specifically address the existential dimension, i.e., how people develop in their search for a meaningful life in the complex and complicated interaction of soma, psyche, and ethos. This “orientation motif,”^2^ Capps states, is central to Erikson’s theory of development. Pastoral care too should aim at helping persons in becoming better oriented in life. The crucial question is: “How does the individual acquire and maintain a sense of orientation in the ongoing process of change?” (Capps 1983, p. 30).

Thus, providing pastoral care consists of assisting people who are disoriented in their life and in the world. Capps distinguishes three causes of disorientation, and concomitantly three roles of the pastor. *The pastor as moral counselor* addresses the loss of moral order; Erikson’s theory of virtues is the underlying frame of this role. *The pastor as ritual coordinator* addresses the experiences that our lives fail to hold together in a meaningful, comprehensible way; here Capps elaborates on Erikson’s theory of ritualization. Finally, *the pastor as personal comforter*, the focus of his 1979 book, addresses the experience of suffering; here Capps pays attention to the negative poles of the psychosocial tasks: mistrust, shame, guilt, etc. He discusses only one of the negative poles, “shame,” to explain the pastor’s role of comforter. As we know, shame is a theme that touched Capps personally, and he would take it up in other publications as both an emerging cultural problem (narcissism and melancholy) and a personal issue with which he wrestled in midlife (Capps 1997, 2015a).

### Deadly Sins and Saving Virtues

In his discussion of the pastor as a moral counselor, Capps uses Erikson’s schedule of virtues to implement this role. It is important to note, he says, that the virtues—hope, will, purpose, etc.—are not abstract capacities but are closely linked to the developmental vicissitudes. Virtues are attitudes or motivational patterns that can be recognized in human behavior, experiences, and queries. Pastors, confronted with the loss of orientation and concomitant suffering, focus on the negative counterpart of the virtues, what Erikson calls “antipathies” or “core pathologies” (e.g., withdrawal linked to hope, compulsion linked to will, inhibition linked to purpose, etc.). Capps introduces here the moral term *vices*. A vice is an “established attitude” or “motivational pattern” (Capps 1983, p. 35) that hinders the developing of virtues. As with virtues, vices too follow a developmental pattern.

For the formulation of the vices, Capps looks to the Christian tradition of the seven deadly sins. In *Deadly Sins and Saving Virtues* (1987) he would present and illustrate in more detail this theory of sins and the concomitant virtues that have a “saving” quality. Sins, he states, are not only “detrimental to human life” but also “contrary to God’s intentions with the world” (Capps 1987, pp. 1ff.). Sin is not to be thought of as “isolated wrongdoings”—an insight to which he returns from an autobiographical perspective in an article on *Young Man Luther* as a classic study (Capps 2015a). Capps explains there how Erikson’s analysis of Luther’s theology on “work” was helpful when, as a young adult, Capps was wrestling with which path to follow in his theological career.

It is evident that Capps is using Erikson’s general theory for designing a theory of *pastoral* (especially *Protestant Christian*) care. That is why he looks for biblical underpinnings of Erikson’s approach and theory. He finds this foundation in the biblical wisdom tradition and in Jesus’ teachings.

## A Biblical Underpinning of Life-Cycle Theory: Natural and Divine Order

In viewing pastoral care as “life orientation,” Capps builds on Erikson’s general existential-psychological terminology intended to grasp the “meaning” of specific human realities in different cultural contexts and epochs. But in *Life Cycle Theory and Pastoral Care*, Capps also offers a theological underpinning of his Eriksonian theory of pastoral care. He notes that there are strong and multiple affinities between Erikson’s developmental theory and the biblical wisdom tradition, especially Proverbs, in terms both of their underlying world view and of specific themes addressed.

First, both speak of practical wisdom as it gets shaped in everyday life. The biblical “wise” were advisers to the king in dealing with the messy business of reigning. Capps suggests that they may be compared to contemporary “advice columnists” (Capps 1983, p. 114)—or, I would add today, TED-talk presenters. Furthermore, like Erikson, the wisdom tradition takes into account the social matrix in which moral and ritual life gets shaped, rather than focusing on abstract philosophical and ethical systems.

Another similarity between Erikson and the wisdom tradition, according to Capps, is that both depart from the conviction that there is an existent “order” in human reality, i.e., in the natural, social, and inner worlds (Capps 1983, ch. 5). Erikson speaks about the “epigenetic principle” or ground plan behind human development; human growth follows a pre-established order according to a universal structure, which means that human development—the psychosocial tasks and related virtues and ritualizations—are each linked to a specific stage of development. As we have seen, the different dimensions (soma, psyche, and ethos) are closely interconnected, and even the life cycles of collective groups of persons hang together, for Erikson, in a “cogwheeling of generations.” In a similar way, the writers of the biblical wisdom tradition discern order in the natural, the social, and the inner worlds (1983, p. 100f) that parallel the divine world. Proverbs favors cause-and-effect reasoning—evil is punished and good results in good (1983, p. 102). In short, there is order in creation, and it is God who has established this order; it is, ultimately, a divine order. This is also the case for Erikson, according to Capps. He states: “Erikson would be very sympathetic to [Maria] Montessori’s view that the developmental process is infused from the beginning with the spirit of God” (1984, p. 121).

Capps takes up the same line of argument in *Deadly Sins and Saving Virtues* (1987). He speaks there of sins as “orientations to life” that are both “detrimental to human life” and “contrary to God’s intentions with the world” (1987, pp. 1ff.). Further, he discerns a connection between the biblical metastory and the structure of the life cycle. The Bible provides “a narrative foundation for the life-cycle as formulated by Erikson” (1987, p. 140). Capps enlarges his biblical foundation by presenting, in addition to the wisdom tradition, the Beatitudes of Jesus and the pilgrimage motive inspired by John Bunyan’s *Pilgrim’s Progress*, the latter of which reappears much later in Capps’s life as a prominent focus of his book *Still Growing* (2015b).

In surveys of laity and clergy that Capps conducted to discover if the “deadly sins” still play a role in contemporary life and, if so, to determine which sins factor most prominently (Capps 1989, 1992; Capps and Cole 2000, 2006),^3^ he retains his focus on the Bible: “The biblical tradition, especially the Beatitudes of Jesus, offers a conception of ‘active faith’ that undergirds the virtues” (2000, p. 360).

This is one of the issues I regret no longer being able to discuss with Capps. Does Erikson indeed emphasize the natural order to such a great extent? And is it possible to force Erikson’s psychological notions into a specifically Christian body of thought?

Let us first look at the role of the natural order in Erikson’s work. I agree that his life-cycle theory definitely has some “universal” ambitions, based as it is on a biological, epigenetic ground plan. The bodily modes of the stages are anchored in embryonic development. But Erikson also stressed that all psychosocial tasks and the related strengths and virtues play a role in every stage. Each virtue is considered “critical,” central for a specific stage, but the later virtues figure in earlier stages in a preliminary form, as anticipations, and the earlier virtues are taken up and further developed again in later stages. The outcome of a developmental stage is not established once and for all. It is a common misunderstanding to consider Erikson’s life-cycle theory an achievement scale: “In each stage the results of the preceding stages are questioned again, and must be reintegrated with regard to the new developmental possibility which is announced. A new integration of all the stage-specific issues is required at each stage” (Zock 1990/2004, p. 34). Moreover, the virtues linked to the five adulthood stages—on which Erikson focuses in his later work—have a much looser connection to bodily development than those of the first three stages. I would argue that the psychosocial tasks and strengths, even in Erikson’s earliest publications, should rather be considered as *existential modes* available to all persons in all times.

The second question I would like to raise with respect to Capps’s biblical underpinning concerns whether contemporary pastoral care should use a model of the self that is anchored in a traditional society. Is not it problematic to adopt the traditional worldview of the biblical wisdom tradition for our time? As Taylor (1989) argues, something has really changed in modernity: the human self is no longer an integral, unquestioned part of a sacred whole. The self has become a “reflexive project” (Giddens 1991). Traditions lose their hold, and individuals have to construct their own identity, appropriating traditions in a personal way. It is the individual who decides if and how a tradition becomes meaningful. Identity, becoming a self, is a construction. I think that Erikson’s psychology—notwithstanding the original universal, biological basis of his life-cycle theory—mainly belongs to the strand of constructivism in identity theory. As I have argued elsewhere, his psychology of religion is basically a hermeneutical one (Zock 1997).

Contemporary identity theorists such as Hubert Hermans even go further. Hermans argues that we need today a model of the self that incorporates postmodern aspects. The autonomous, reflexive self of modernity, in charge of giving meaning to one’s own life, is deconstructed in the global, postmodern context; the self has become multiple, fragmented, and decentered under the influence of continually changing and diverse cultural forces (Hermans and Hermans-Konopka 2010; Zock 2011, 2013). In Hermans’s view, this does not mean that traditional and modern models of the self have become completely obsolete. He notes that aspects of the traditional and the modern self are still present in postmodern selves: postmodern persons still need and may experience “being part of the a wider, cosmic order” and involved in a “wider whole” (Hermans and Hermans-Konopka 2010, pp. 163, 166; Zock 2013, pp. 21ff.). “Faith as emanating from the traditional self contributes to a sense of continuity and stability of the self needed to survive in a period in which change, flux, discontinuity and uncertainty are more prominent than ever before” (Hermans and Hermans-Konopka 2010, p. 99).

In my view, this makes it possible that biblical texts such as Proverbs and the Beatitudes may remain an *inspirational* source for pastoral care even when one does not necessarily share or assume their worldview. But there is a difference. In order to feel part of a greater whole, some sort of personal appropriation is necessary. Hermans speaks about “individualized spirituality” as the current mode of incorporating traditional religion.^4^ Cultural and religious traditions, and their representatives such as pastors, no longer possess matter-of-fact authority.

## Beyond a Biblical Underpinning? Postmodern Aspects of Capps’s Later Work

Capps did not explicitly address the issue of postmodern development of the self and its consequences for pastoral care. However, his work shows a sensitivity in this respect. His thinking about the coherence of natural and divine order may not have been rigid, and his views on the “orderly” foundation of development definitely change in his later work. In *Deadly Sins and Saving Virtues,* we already find some indication of this. Capps states there that the narrative biblical metastructure is an expression of the “divine order,” but in fact he is using biblical stories and concepts in a literary and contemporary way. For instance, he looks at the experiences of Bunyan’s pilgrim, Christian, through the lens of the life cycle (school age and adolescence) and uses the imagery of pilgrim in talking about the Bible: “The pilgrimage begins in the expulsion from the Garden of Eden and culminates in the fields, or perhaps on the threshing floor, of Boaz. The pilgrimage from Genesis to Ruth portrays the victory of the saving virtues over the deadly sins. And like the blessed assurances of Jesus’ Beatitudes, the story has the power to create the very restitution it portrays” (Capps 1987, p. 142).

In addition, in his empirical surveys on the deadly sins Capps suggests that traditional religious language may not be appropriate for speaking about contemporary life attitudes.^5^ He chooses not to use the traditional names of the sins in his questionnaire but instead gives a contemporary behavioral description of them. His stated rationale for not including the traditional names of the sins and virtues was a concern that even the words “sin” and “virtue” might prompt respondents to react to the identifying terms rather than to the description itself. By including descriptions only, there was greater assurance of “a common understanding” of what the item meant (1987, p. 365). Thus, Capps describes greed in the survey as “a consuming desire for wealth or affluence, causing one to think of little else,” and pride as “a self-centered attitude where one is continually expecting or demanding praise and adulation” (Capps and Cole 2006, p. 523).

Some 20 years later, in his publications on the decades of life (Capps 2004, 2008), we see a radical change in Capps’s use of the life-cycle theory. In place of Erikson’s eight psychosocial stages and related life-periods, Capps conceives of the life cycle in terms of ten decades of life. The virtue of hope is now no longer linked to infancy but to childhood (ages 0 through 9), the virtue of will no longer to the age of toilet training and learning to walk but to the teenage years (ages 10 through 19), and so on. We must note that Capps abandons here the developmental pattern according to the epigenetic ground plan and hence also any anchorage in the “natural order” he previously saw as intrinsic to both Erikson’s life-cycle theory and his own theory of pastoral care.

Another significant change in this new developmental model is that Capps—at that point himself almost a septuagenarian—shifts his focus to adult development. Six (in contrast to Erikson’s original three) stages for Capps here focus on the adult, three of these on older adulthood. Capps likewise adds two psychosocial issues he attributes to very old age: *release versus control*, resulting in the virtue of *grace* for the ninth decade; and *desire versus struggle*, resulting in the virtue of *endurance*, for the tenth decade. Here, we see that Capps builds further on the theory of the life cycle, taking into account both the changing sociocultural context—people are living much longer today than 80 years ago—and his own experiences with aging.

In the new ten-decades model, development no longer culminates in “wisdom,” the virtue of Erikson’s last stage and now of Capps’s eighth decade. Is it a coincidence that the biblical wisdom tradition figures less in *The Decades of Life*? Capps’s point of reference is no longer Proverbs; instead, Ecclesiastes is quoted time and again. Could this be because the latter biblical book better matches a postmodern sense of the relativity of human existence? It is striking that Capps, even more than in earlier writings, freely and loosely uses different cultural sources to explain his new model of development and to illustrate the individual case-histories he presents—not only the Psalms and Bunyan’s *Pilgrim’s Progress*, but also Shakespeare, poems, and nursery rhymes. I also observe that, surprisingly, any reference to “sins” is absent in *The Decades of Life*. Is this because Capps’s focus shifted to positive aspects of development? Or did he perhaps consider the traditional language of sins, associated narrowly with specific religious traditions, as less appropriate in the present time?

So we may conclude that over time Capps gradually abandoned the idea that his pastoral care model needed a biblical underpinning in the natural and divine order. Instead, he adopts a rather phenomenological, almost literary, and, I would say, postmodern approach, freely using inspirational sources. This approach comes even more to the fore in *Still Growing* (2015b), a quite personal, artistic, and creative account of life issues in which he returns once again to two foundational sources of his inspiration, Sigmund Freud and William James. He seems to have arrived here at a rather positive view of human development, one somewhat at odds with a skeptical, relativistic, postmodern attitude. Capps argues in *Still Growing* that growth is possible even, or especially, in very old age. This positive angle is also visible in his pastoral theology when, for instance, he describes the pastor as “an agent of hope” (Capps 2005) and in the title of one of his last books, *The Resourceful Self* (Capps 2015c).

In these ways Capps once again returns to embrace Erikson’s optimistic, one might say modern, view of human development. Although individuals may get stuck in the negative poles of mistrust, shame, and guilt, the stages of development later in life bring new capacities, contexts, and social relations. With new challenges come more opportunities for growth. Similarly, the last psychosocial-existential task and ideal aim of development in Erikson’s view is integrity. It is this last stage that exemplifies how individual lives are connected in the cycle of generations. Erikson beautifully captures this sense in his well-known saying that if old people are not afraid to die, young people will not be afraid to live.

## Taking Stock: Donald Capps and Postmodern Pastoral Care

The processes of globalization and secularization have only multiplied in significance since Donald Capps published his *Life Cycle Theory and Pastoral Care* in 1983. Pastors and chaplains (at least in Western countries) are ministering to and with individuals from diverse ethnic, religious, and racial backgrounds in a confusing secular environment. At the same time, pastoral authority is often questioned and the influence of religious institutions is declining. It is no longer routinely accepted that wisdom “resides in the community, not in the individual” (Capps 1983, p. 115). These developments have had huge consequences for pastors. The number of conflicts between ministers and their congregations appears to be increasing along with burn-out of pastoral professionals. The vocational profile of the pastor demands new articulation and appropriation. What today is the representative function of the pastor? What is unique to the work of pastoral guidance in comparison, for instance, to that of psychologists and social workers? What remains of the pastor’s role of ritual coordinator now that it is no longer readily accepted that pastors (or laypersons) “personify the ritual wisdom of the community” (Capps 1983, pp. 115ff.)? In the Netherlands, we see many pastors involved in ritual guidance and support outside the churches and congregations. New ritual practices are being created (for marriage and divorce, for life situations such as death and grieving, and for birth and graduation), mixing traditional and new elements. The pastoral profession is on the move in the fluid global religious field.

I have drawn attention to a development in Capps’s writing and thinking of an increasing postmodern sensitivity. He started by seeking a biblical-theological justification for his developmental model of pastoral care as one reflecting a natural and divine order. However, he gradually began to use the theory more freely in literary, hermeneutic, and personal ways. In my view, it is precisely because of its focus on “meaningful” aspects of psychosocial development that Erikson’s life-cycle theory remains a powerful instrument for pastoral care today (see Zock 2011). Speaking of sins and virtues in a general, non-Christian sense, as Capps did in his surveys on deadly sins and saving virtues and in *The Decades of Life*, is useful in offering guidance to and with secular persons in a postmodern age. This vocabulary may resonate with both religious and non-religious people because it addresses basic human existential themes and challenges (see Zock 2015). While traditional religious languages and traditions may (and do) become obsolete, their underlying infrastructures of meaning find ever new expressions in the language and practices of contemporary forms of lived religion. Spiritual caregivers are developing overarching spiritual languages and models to adequately address these.^6^

There are many questions in this respect I would have loved to discuss with Donald Capps. How would he integrate ideas about the multi-voiced, fragmented, postmodern self in his theory of pastoral care? How might he assess new “pastoral services” outside the churches? How should cooperation in multidisciplinary care teams take shape? Would he agree with the demand for evidence-based, social-scientific research on what pastors actually do and in what their contribution to healthcare consists? Is a literary hermeneutic approach enough for the present-day pastor?

In my view, not only a psychological but also a social, cultural, and historical multidisciplinary analysis should be part of pastoral theology in a postmodern world. The sociocultural context—what Erikson called *ethos*—should be structurally addressed in order to understand existential and religious developments in our time. But the personal and artistic ways in which Capps freely uses his own experiences and religious tradition is a wonderful example of postmodern life and scholarship. Fixed anchors no longer exist, and biblical as well as other cultural traditions have become personal sources of inspiration rather than guiding frameworks. For me, Erikson’s life-cycle theory, if seen as a general, hermeneutic, contextual model for analyzing the existential dimensions of human life and development and as a tool of cultural analysis, is of enormous value in the postmodern context. The work of Donald Capps helped me to better understand the intricate and often hidden religious dimensions of meaning-making in developmental processes and cultural phenomena. His expansive thinking, resourcefulness, and creativity in developing ways to address deeply existential dimensions of human life will be an enduring source of inspiration to me.

## Footnotes

1 “Erikson’s writings in the 1970s are those of a man who had come out of the closet and was seeing the reality around him less as a professional psychoanalyst and more as a man whose outlook was unabashedly religious in tone and scope” (Capps 1996, p. 327).

2 “… the importance of having a clear sense of orientation in life, a steady image of where we have been and where we are going” (Capps 1983, p. 30).

3 “The survey was developed in response to some scepticism (expressed by students and others) that the traditional formulation of the deadly sins is viable for late twentieth-century America. Does this formulation really get at contemporary Americans’ sense of dis-ease, their inner sense of wrongness, their sense of being inherently flawed? The only way to respond to such doubts would be, it seemed, to put this matter to empirical test” (Capps and Cole 2000, p. 360). Capps and Cole concluded that the descriptions of the sins are still very relevant.

4 Hermans sees contemporary tendencies to religious fundamentalism as emotional, defensive counterreactions to the ontological insecurity of the postmodern self in a global world (Zock 2013, p. 20).

5 Capps states that he wanted “to adapt the traditional deadly sins model to contemporary understandings of the attitudes that are destructive of self and others” (Capps and Cole 2006, p. 520). This implies the need for a different language.

6 An example is the Spiritual Care Guideline recently developed in the Netherlands: http://www.oncoline.nl/index.php?pagina=/richtlijn/item/pagina.php&id=36577&richtlijn_id=907.
